# Assessment of the relationships between myocardial contractility and infarct tissue revealed by serial magnetic resonance imaging in patients with acute myocardial infarction

**DOI:** 10.1007/s10554-015-0678-y

**Published:** 2015-06-06

**Authors:** Christie McComb, David Carrick, John D. McClure, Rosemary Woodward, Aleksandra Radjenovic, John E. Foster, Colin Berry

**Affiliations:** Clinical Physics, NHS Greater Glasgow and Clyde, Glasgow, UK; BHF Glasgow Cardiovascular Research Centre, University of Glasgow, 126 University Place, Glasgow, G12 8TA UK; Department of Cardiology, Golden Jubilee National Hospital, Clydebank, UK; Department of Diagnostic Imaging, Golden Jubilee National Hospital, Clydebank, UK

**Keywords:** Myocardial infarction, Strain-encoded CMR, Contractility

## Abstract

Imaging changes in left ventricular (LV) volumes during the cardiac cycle and LV ejection fraction do not provide information on regional contractility. Displacement ENcoding with Stimulated Echoes (DENSE) is a strain-encoded cardiac magnetic resonance (CMR) technique that measures strain directly. We investigated the relationships between strain revealed by DENSE and the presence and extent of infarction in patients with recent myocardial infarction (MI). 50 male subjects were invited to undergo serial CMR within 7 days of MI (baseline) and after 6 months (follow-up; n = 47). DENSE and late gadolinium enhancement (LGE) images were acquired to enable localised regional quantification of peak circumferential strain (Ecc) and the extent of infarction, respectively. We assessed: (1) receiver operating characteristic (ROC) analysis for the classification of LGE, (2) strain differences according to LGE status (remote, adjacent, infarcted) and (3) changes in strain revealed between baseline and follow-up. 300 and 258 myocardial segments were available for analysis at baseline and follow-up respectively. LGE was present in 130/300 (43 %) and 97/258 (38 %) segments, respectively. ROC analysis revealed moderately high values for peak Ecc at baseline [threshold 12.8 %; area-under-curve (AUC) 0.88, sensitivity 84 %, specificity 78 %] and at follow-up (threshold 15.8 %; AUC 0.76, sensitivity 85 %, specificity 64 %). Differences were observed between remote, adjacent and infarcted segments. Between baseline and follow-up, increases in peak Ecc were observed in infarcted segments (median difference of 5.6 %) and in adjacent segments (1.5 %). Peak Ecc at baseline was indicative of the change in LGE status between baseline and follow-up. Strain-encoded CMR with DENSE has the potential to provide clinically useful information on contractility and its recovery over time in patients with MI.

## Background

Regional left ventricular (LV) systolic function has prognostic importance in survivors of myocardial infarction (MI) [1, 2]. Imaging of LV wall motion is an indirect method for assessing contractility, and cardiac magnetic resonance (CMR) can now provide information on LV contractility using strain-specific techniques. Measurement of strain rather than LV motion should theoretically increase the diagnostic accuracy and prognostic value of cardiac imaging in patients with a history of MI [3]. The fact that CMR provides quantitative information on infarct pathology as well as myocardial strain makes this imaging modality attractive for clinical and research purposes in patients with a history of MI.

During the cardiac cycle, the heart moves through and rotates within a given imaging plane. The relationship between the apparent motion of the endocardial and epicardial borders and the contraction and relaxation of the myocardium is complex, and a true assessment of contractile function requires tracking of discrete points within the myocardium [4]. Three types of magnetic resonance imaging (MRI) pulse sequence which have previously been used for the visualisation and quantification of myocardial deformation are tagging [5–8], velocity-encoded phase contrast imaging [9–12] and strain-encoded (SENC) imaging [13].

The main drawbacks of myocardial tagging and SENC techniques are that the need to apply a saturation grid limits the resolution, and quantitative analysis can be complicated and time consuming. Velocity-encoded phase contrast methods are sensitive to accumulated errors that can result in inaccuracies in position measurements, and displacements can only be measured over short periods of time.

Displacement ENcoding with Stimulated Echoes (DENSE) is an alternative method for the quantification of myocardial strain [14, 15]. Stimulated echoes are used to provide a high spatial density of displacement measurements in the myocardium over long time intervals, which enable large displacements to be encoded. Since displacement is directly encoded in the phase, there is no requirement for tag detection and analysis is less time-consuming than for tagging.

Much of the research performed with DENSE so far has been methods development, and its diagnostic value in a clinical setting is uncertain. Recently, Miyagi et al. [16] reported that circumferential strain revealed by DENSE is associated with the presence of late gadolinium enhancement (LGE). However, the relationships between regional LV contractility and infarction are not uniform. Regional contractility may be influenced by the spatial proximity to the infarct tissue, which itself may change in composition and size according to the time interval from the acute MI event [17]. Therefore, we aimed to assess the relationships between regional myocardial contractility revealed by DENSE and LGE in patients with acute MI undergoing serial imaging.

## Methods

### Study population

Fifty male patients (age 56 ± 10 years) underwent CMR within 7 days of ST-elevation myocardial infarction (STEMI), and were invited to return for a follow-up scan after 6 months. All patients underwent revascularization with primary percutaneous coronary intervention (PCI) in the Golden Jubilee National Hospital, according to contemporary standards of care.

The research protocol was approved by the regional Research Ethics Committee and written informed consent was obtained from each subject.

### Cardiac MRI

All images were obtained using a 1.5 T Siemens Avanto MRI scanner (Siemens, Erlangen, Germany) with a 6-channel phased-array body coil (anterior) and an 8-channel phased-array spine coil (posterior). The CMR protocol included DENSE imaging for the assessment of strain and LGE imaging in a co-registered mid-ventricular short axis slice, positioned at mid-papillary level. Cine imaging was also acquired to enable quantification of LV dimensions and function.

DENSE imaging parameters were as follows: echo time 8 ms; repetition time 16.3 ms; flip angle 20°; slice thickness 8 mm; field of view 360 mm × 270 mm; matrix size 112 × 84; displacement encoding of 0.2 π/mm; EPI factor of 8.

Early gadolinium enhancement (EGE) imaging was acquired 1, 3, 5 and 7 min post-contrast injection using a TrueFISP readout and fixed inversion time (TI) of 440 ms. Late gadolinium enhancement images covering the entire LV were acquired 10–15 min after IV injection of 0.15 mmol/kg of gadoterate meglumine (Gd^2+^-DOTA, Dotarem, Guebert S.A., Villepinte, France) using segmented phase-sensitive inversion recovery (PSIR) turbo fast low-angle shot sequence in all cases [18]. LGE imaging parameters were as follows: echo time 3.4 ms; repetition time 8.7 ms; flip angle 20°; slice thickness, 8 mm; field of view 340 mm × 270 mm; matrix size 256 × 156. The voxel size was 1.8 × 1.3 × 8 mm^3^. A Look-Locker TI scout scan was undertaken to determine the inversion times associated with optimal nulling of the myocardial signal. The inversion times were in the range of 260–350 ms.

Cine images were acquired using a b-SSFP sequence with the following parameters: echo time 1.2 ms; repetition time 3.3 ms; flip angle 70°; slice thickness 7 mm; field of view 340 mm × 270 mm; matrix size 256 × 180.

The imaging protocol was the same for all patients for the baseline and follow-up CMR scans.

### Image analysis

The images were analysed on a Siemens work-station by observers with at least 3 years CMR experience (C.M., D.C.). The LGE and DENSE analysis were performed independently by two trained operators (DC, CM) who were blinded to the results of each other’s findings.

#### Analysis of left ventricular volumes and ejection fraction

Left ventricular dimensions, volumes and ejection fraction were quantified using computer-assisted planimetry (syngo MR^®^, Siemens Healthcare, Erlangen, Germany). Endo- and epi-cardial borders were manually delineated on the cine images, and LV dimensions and systolic function (ejection fraction) were measured with the automated analysis software. The LV was segmented using the anterior right ventricular-LV insertion point as the reference point.

#### Analysis of strain-encoded MRI with DENSE

Each of the short axis DENSE images were divided into six segments according to the American Heart Association (AHA) model in order to standardise the approach to regional analysis of LV strain [19]. Endocardial and epicardial borders were contoured and strain values were measured on a per-segment basis.

DENSE images were analysed using CIM_DENSE2D software (University of Auckland, Auckland, New Zealand) [20], and for each myocardial segment, values were obtained for peak circumferential strain (Ecc) (%).

#### Analysis of late gadolinium enhancement MRI

The presence of acute infarction was established based on abnormalities in cine wall motion, rest first-pass myocardial perfusion, and delayed-enhancement imaging in two imaging planes. LGE image analysis was performed using Argus software (Siemens, Erlangen, Germany). Each of the short axis LGE images were divided into six segments according to the AHA model [19]. A region of interest (ROI) containing at least 100 pixels was drawn in an area of remote myocardium, and a threshold level was set at the mean value of the ROI plus 5 standard deviations (SD) [21]. The territory (area) of infarction was delineated manually using computer-assisted planimetry and based on the 5 SD threshold above the remote reference region ROI. The myocardial mass of late gadolinium (grams) was quantified and expressed as a percentage of total LV mass.

#### Tissue categorisation: designation of zones of tissue based on pathology

The terms ‘remote’, ‘infarct’ and ‘adjacent’ were used to reflect areas of myocardial tissue that were defined by the presence (‘infarct’ and ‘adjacent’) or absence (‘remote’) of MI pathology, as revealed by LGE on MRI. The remote myocardium was defined as myocardium 180° from the affected zone with no visible evidence of infarction or wall motion abnormalities (assessed by inspecting corresponding contrast enhanced LGE and cine images, respectively).

### Statistical analysis

All analyses were performed using Minitab 16 (Minitab Inc, PA, USA), with the exception of ROC analysis, which was performed using SPSS 19 (IBM, New York, USA).

Prior to performing statistical analysis, all data were checked for normality using Anderson–Darling tests. Peak Ecc was found to be non-normally distributed, and analysis was therefore performed using non-parametric tests.

#### ROC analysis, sensitivity and specificity

ROC analysis was performed based on the presence or absence of LGE within each segment, at both baseline and follow-up. Thresholds for peak Ecc were established by finding the values which corresponded to the maximum average sensitivity and specificity.

A score was allocated to each segment according to the transmural extent of infarction (percentage of LGE) within the segment (0: 0 %, 1: 1–25 %, 2: 26–50 %, 3: 51–75 %, 4: 76–100 %). For each score, the percentage of segments which were correctly identified as containing LGE (scores 1–4) or not containing LGE (score 0) was calculated.

#### Comparison with LGE Status

At both baseline and follow-up, segments were classified into three groups depending on LGE status: infarcted, adjacent (infarction in one or more adjacent segments) and remote (no infarction in adjacent segments). Strain values in the three groups were compared using a Kruskal–Wallis test along with individual Mann–Whitney tests.

#### Assessment of longitudinal changes

For segments categorised as remote, adjacent and infarcted at the baseline scan, Wilcoxon signed rank tests were used to compare the baseline and follow-up results for each category separately. A Kruskal–Wallis test along with individual Mann–Whitney tests was then used to compare the strain differences (follow-up—baseline) between the three categories.

The data were then further categorised according to the change in LGE status between baseline and follow-up e.g. remote (baseline) → remote (follow-up), remote (baseline) → adjacent (follow-up), adjacent (baseline) → infarcted (follow-up) etc. Segments which were categorised as remote, adjacent and infarcted at baseline were considered separately, and the changes in strain (follow-up—baseline) between sub-categories [e.g. remote (baseline) → remote (follow-up) vs remote (baseline) → adjacent (follow-up)] were compared using Kruskal–Wallis with individual Mann–Whitney tests. A similar comparison was then performed to assess if differences in strain values obtained at baseline could be detected between sub-categories.

A Bonferroni correction for multiple testing was used with non-parametric tests including the Kruskal–Wallis and Mann–Whitney tests. A *p* value of 0.05 was adopted to reject the null hypothesis of no difference.

## Results

**Fig. 1:**
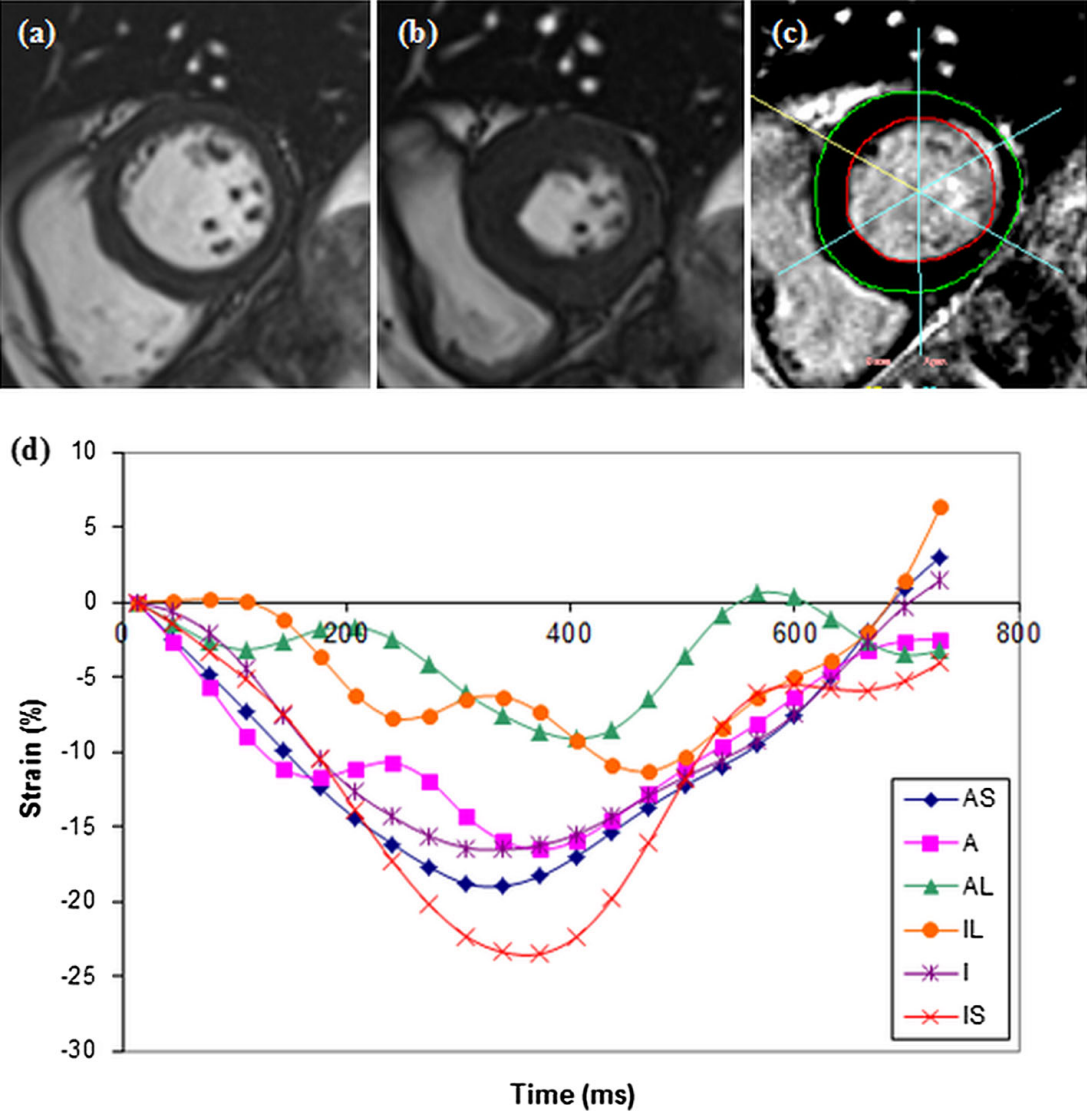
Cine images at **a** end diastole and **b** end systole showing a wall motion defect in the anterolateral and inferolateral segments, and **c** the corresponding LGE image. **d** Graphical representations of circumferential strain throughout the cardiac cycle, as determined by DENSE. *AS* antero-septal, *A* anterior, *AL* antero-lateral, *IL* inferolateral, *I* inferior, *IS* inferoseptal

Table 1 summarises the clinical characteristics of the STEMI patients and LV function and volumes as determined by cine CMR. The images obtained from a 70 year-old male with an acute sub-endocardial scar in the lateral left ventricular wall are shown in Fig. 1.

**Table 1:** Characteristics of STEMI patients (n = 50)

Characteristic	
Age (years)	56 ± 10 (33–80)
BMI	28 ± 4 (20–38)
Smoker	38 (63 %)
Hypertension	11 (18 %)
Hypercholesterolaemia	10 (17 %)
Prior myocardial infarction	1 (2 %)
Culprit coronary artery	
Left anterior descending	19 (38 %)
Left circumflex	9 (18 %)
Right	22 (44 %)
Electrocardiogram	
Q-waves	25 (50 %)
Troponin T (ng/l)	3353 (2005, 6625)
LVEF (%)	
Baseline	54 ± 9 (35–70)
Follow-up	62 ± 8 (40–76)
LVEDV (ml)	
Baseline	153 ± 33 (85–231)
Follow-up	163 ± 69 (92–251)
LVESV (ml)	
Baseline	72 ± 23 (35–119)
Follow-up	64 ± 26 (23–150)

Age, BMI, LVEF, LVEDV and LVESV are expressed as mean ± SD (range). Troponin T is expressed as median (interquartile range). The electrocardiogram (ECG) was obtained during the index hospitalisation

*BMI* body mass index, *LVEF* LV ejection fraction, *LVEDV* LV end-diastolic volume, *LVESV* LV end-systolic volume

From the 50 STEMI patients who underwent baseline scans, 47 attended for follow-up scans, and DENSE images from 4 of these patients were considered non-diagnostic due to breathing artefacts. This gave totals of 300 and 258 segments which were available for analysis at baseline and follow-up respectively. LGE was present in 130/300 (43 %) and 97/258 (38 %) segments, respectively.

### ROC analysis, sensitivity and specificity

**Fig. 2:**
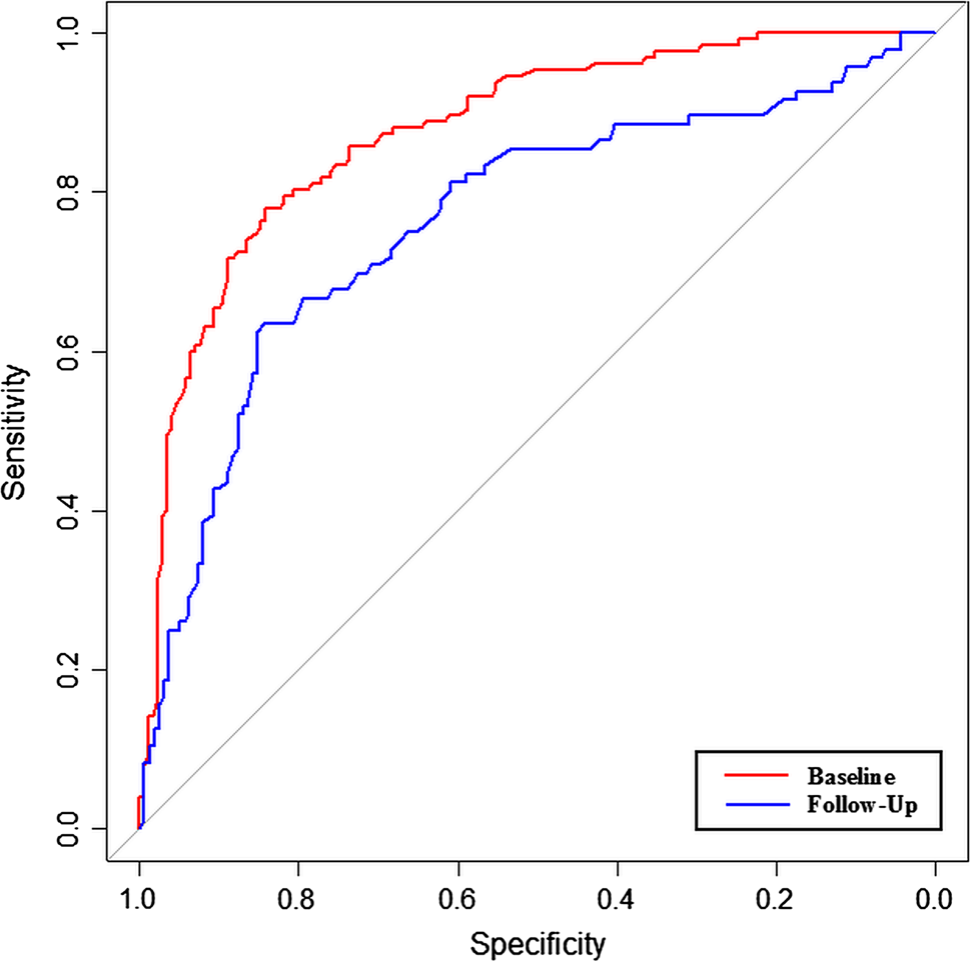
ROC curves for the presence of infarction using DENSE strain parameters at baseline and 6 month follow-up

The ROC curves for strain-encoded CMR with DENSE and the classification of LGE (present/absent) on a per-segment basis at baseline and at follow-up are shown in Fig. 2.

The peak Ecc strain threshold, area-under-the-curve (AUC), sensitivity and specificity for the classification of contractile abnormalities associated with the presence of LGE at baseline and follow-up are shown in Table 2. The AUC for detection of infarct tissue (LGE) by DENSE was 0.88 on baseline scans and 0.76 at follow-up. The percentage of segments which were correctly identified as containing no LGE (score 0) and containing LGE (scores 1–4) are shown in Table 3. The percentage of segments that were correctly identified at baseline and at follow-up was greatest for scores LGE scores of 0 (no LGE) and 4 (100 % transmural extent of LGE). Further investigation of the non-infarcted segments (score 0) which were incorrectly classified by DENSE as containing LGE showed that 61 and 52 % were located adjacent to segments which contained LGE at baseline and follow-up respectively.

**Table 2:** Threshold, sensitivity, specificity and AUC of peak circumferential strain (Ecc) as measured by DENSE for the detection of infarct tissue revealed by late gadolinium enhancement

	Threshold (%)	Sensitivity (%)	Specificity (%)	AUC
Baseline	12.8	84	78	0.88
Follow-up	15.8	85	64	0.76

**Table 3:** Percentage of segments which were identified as containing late gadolinium enhancement (LGE, transmural extent scores 1–4) or not containing LGE (score 0) using the thresholds for peak circumferential strain (Ecc)

	Score 0	Score 1	Score 2	Score 3	Score 4
Baseline	84	63	79	88	93
Follow-up	84	40	72	75	93

### Comparison of myocardial strain with the presence and transmural extent of LGE

**Fig. 3:**
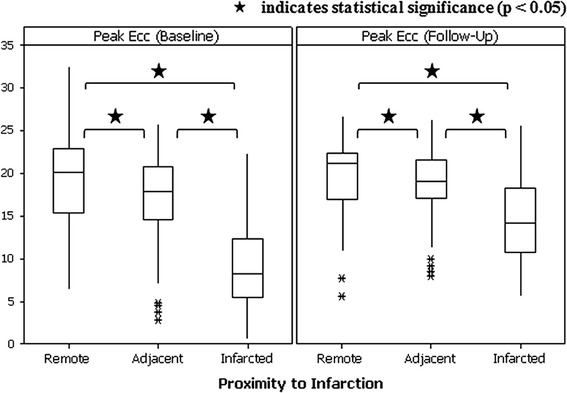
Comparison of peak Ecc with segments categorised according to LGE status at baseline and at follow-up

Differences were observed for peak Ecc measured in infarcted segments versus peak Ecc in both remote and adjacent segments, and between peak Ecc in remote and adjacent segments, at both baseline and follow-up. The results are illustrated in Fig. 3.

### Relationships between myocardial strain and LGE revealed by serial imaging after acute MI

From the 43 patients who attended for both baseline and follow-up scans, 122 segments had LGE at baseline and/or at follow-up.

A comparison of the peak Ecc strain values obtained at baseline and follow-up found differences in segments classified as adjacent and infarcted, but not in remote segments. An increase in peak Ecc was observed in both adjacent and infarcted segments, with median differences (follow-up—baseline) of 1.5 and 5.6 % respectively.

**Fig. 4:**
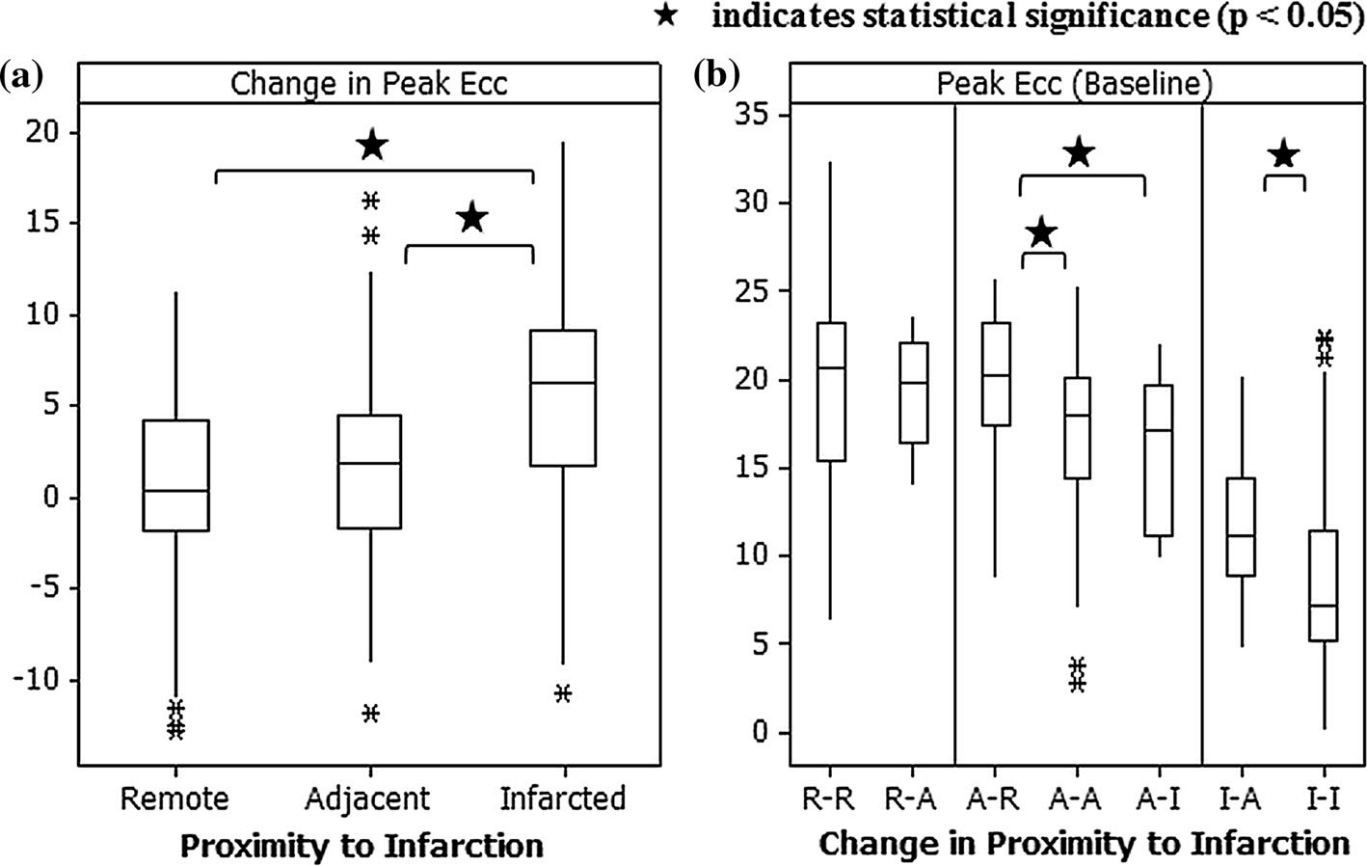
Comparison of **a** change in peak Ecc with segments categorized according to LGE status (infarct, adjacent, remote) at baseline and **b** peak Ecc at baseline with segments categorised according change in LGE status (infarct, adjacent, remote) reflecting post-MI remodelling between baseline and follow-up

The results of the Kruskal–Wallis test with individual Mann–Whitney tests comparing the three categories of tissue pathology (remote, adjacent, infarcted) are illustrated in Fig. 4a. Differences were observed between the change in strain in remote and infarcted segments, and between adjacent and infarcted segments.

Within each designated pathological tissue category (remote, adjacent, infarcted), comparison of tissue categories showed no statistically significant differences when considering the change in strain between baseline and follow-up. However, differences were observed when comparing baseline strain between sub-categories, as illustrated in Fig. 4b.

**Fig. 5:**
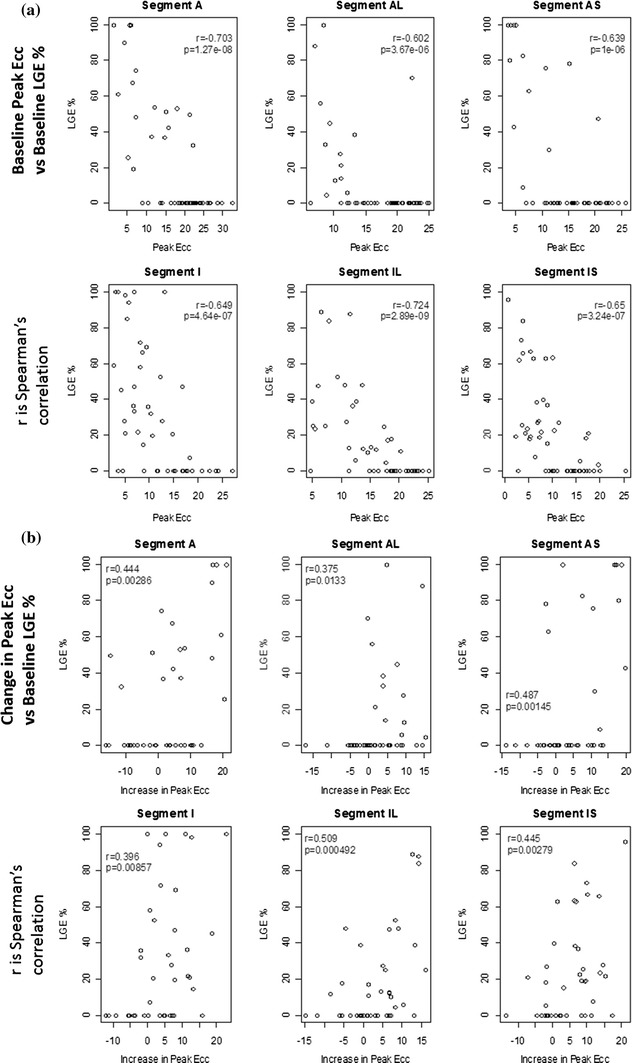
**a** Relationship between peak Ecc versus transmural extent of infarction (%) at baseline on a per segment basis at the mid-ventricular level. Peak Ecc is moderately well correlated with the transmural extent of infarction (%) with a high level of statistical significance (*p* < 0.0001); **b** Relationship between change in peak Ecc at follow-up versus baseline versus transmural extent of infarction (%) at baseline on a per segment basis at the mid-ventricular level. Change in peak Ecc is correlated with the transmural extent of infarction (%). The relationships between strain and the transmural extent of infarction are consistent across the different myocardial regions

On a per-segment basis at the mid-ventricular level, peak Ecc (Fig. 5a) and change in peak Ecc at follow-up from baseline (Fig. 5b) correlated moderately well with the transmural extent of infarction at baseline, and these relationships were consistently similar for each of the anatomical regions.

## Discussion

The results of this investigation have shown that, firstly, in line with previous studies [17], the number of segments with evidence of LGE is less at 6 months compared with 2 days post-MI. Secondly, peak myocardial circumferential strain as measured by DENSE has high discriminative value between healthy myocardium and infarct tissue as revealed by LGE. Thirdly, this relationship was observed within the first week after STEMI and again at a follow-up scan 6 months later, and it was influenced by the time from the index event, the proximity of the segment to the infarct territory, and the transmural extent of MI. Finally, the change in peak circumferential strain over time i.e. strain recovery is associated with the transmural extent of infarction at baseline.

The standard CMR assessment of LV function in patients with a history of MI involves using cine images to measure global parameters such as LV end-systolic volume, LV end-diastolic volume and LV ejection fraction. However, most pathologies do not affect the heart uniformly, and regional dysfunction may be masked by seemingly “normal” values of global function [22]. Assessment of regional myocardial function therefore has an important role to play in the diagnosis and management of cardiac disorders, and in determining the long-term prognosis for these patients, including in patients with a history of MI.

In a study which used strain measurements obtained with SENC to compare myocardial segments with non-transmural and transmural infarction, with transmural infarction defined as >75 % hyperenhancement in LGE imaging, an optimal threshold for peak Ecc of 10 % was identified, with a corresponding sensitivity of 97 %, specificity of 94 % and AUC of 0.96 [23]. A similar study which used tagging to compare non-infarcted and infarcted segments identified a threshold for peak Ecc of 20 %, with a corresponding sensitivity of 92 % and a specificity of 99 % [3]. More recently, Miyagi et al used ROC analysis to compare non-infarcted and infarcted segments, and obtained an AUC value of 0.92 for peak Ecc [16]. In this study, thresholds for peak Ecc of 13 and 16 % were identified at baseline and follow-up respectively. The corresponding sensitivity, specificity and AUC values shown in Table 2 were lower than those obtained in other studies, but were still considered to be high, particularly at baseline.

The higher threshold value identified at follow-up compared to baseline indicates that the presence of LGE at follow-up may be associated with a lower degree of contractile dysfunction, however the relationship between peak Ecc and LGE may be complicated by the complex remodelling process. In a patient with recent MI, the injured territory revealed by LGE may also contain oedema and viable myocardium amenable to salvage [17]. The potential for salvage is influenced by clinical factors, such as the timing and success of coronary revascularization [24]. Therefore, the presence of LGE in a patient with recent MI may be associated with functional recovery. Alternatively, the persistence of chronic LGE at follow-up is typical of permanent infarction although the extent of infarction typically falls over time reflecting infarct remodelling and salvage of acutely jeopardised myocardium in some segments, but not in other segments. Given the complexities of infarct remodelling and salvage, direct measurement of regional LV contractility could provide further information on the potential for recovery or not, for clinical and research purposes.

Differences in peak circumferential strain between remote and infarcted segments can be detected using tagging [25]. The results of this study have shown that measurements of peak Ecc obtained using DENSE can additionally reveal differences between remote and adjacent segments, not only at baseline but also at 6 months post-MI.

We observed that when segments were sub-categorised according to the change in LGE status between baseline and follow-up, there were differences in baseline strain between sub-categories for adjacent and infarcted segments. Higher strain values were obtained in adjacent segments which would subsequently be re-classified as remote, and lower strain values were obtained in segments which would be re-classified as infarcted. Infarcted segments which would subsequently be re-classified as adjacent had higher strain than those which remained infarcted. Strain measurements in adjacent and infarcted segments at baseline may have prognostic value relating to the progression or recovery of contractile abnormalities in the chronic setting.

### Limitations

The results of this study show that DENSE has the potential to provide clinically useful information in patients with recent and chronic MI. However, the limitations in the design should be noted. Only one mid-ventricular slice was obtained for each participant, and the analysis method does not take adjacent segments and/or slices into account. The implementation of DENSE available at our institution does not account for through-plane motion of the myocardium during image acquisition; however 3D versions of the DENSE pulse sequence have been developed [26–28] and could be assessed in future studies.

## Conclusions

DENSE has the potential to provide clinically useful information relating to contractile abnormalities associated with the presence of LGE, and the recovery of contractile function over time. Continuing developments in the technique, including 3D versions which account for through-plane motion, will increase the applicability in the clinical setting.
